# Formation of potentially toxic carbonyls during oxidation of triolein in the presence of alimentary antioxidants

**DOI:** 10.1007/s00706-017-2036-3

**Published:** 2017-10-19

**Authors:** Marini Damanik, Michael Murkovic

**Affiliations:** Institute of Biochemistry, Graz University of Technology, Graz, Austria

**Keywords:** Triolein, Lipid oxidation, Aldehydes, DNPH, Antioxidants, β-Carotene, α-Tocopherol

## Abstract

**Abstract:**

A relation between oil uptake and cancer as well as induction of hepatic inflammation was shown earlier. It is discussed that the main oil oxidation products—hydroperoxides and carbonyls—might be the reason for the mentioned diseases. In this manuscript quantitative determination of aldehydes which are formed during oxidation of triolein—as a model substance—using the Rancimat 679 is described. The oxidation of 11 g of triolein is carried out at 120 °C sparging air with a flow of 20 dm^3^/h for 10 h. A series of aliphatic aldehydes starting from hexanal to decanal as well as decenal was identified by LC–MS/MS and quantified as DNPH derivatives. In addition, the total amount of carbonyls was determined. Based on the calibration with hexanal, all other dominant substances were in the similar concentration range with maximum concentrations of 1.6 µmol/cm^3^ of hexanal, 2.3 µmol/cm^3^ of heptanal, 2.5 µmol/cm^3^ of octanal, 3.2 µmol/cm^3^ of nonanal, 4.0 µmol/cm^3^ of decanal after 6 h. The total amount of carbonyls reached a maximum after 6 h being 27 µmol/cm^3^ for triolein without antioxidant. The results of this investigation will be a basis for further toxicological studies on oxidized oils.

**Graphical abstract:**

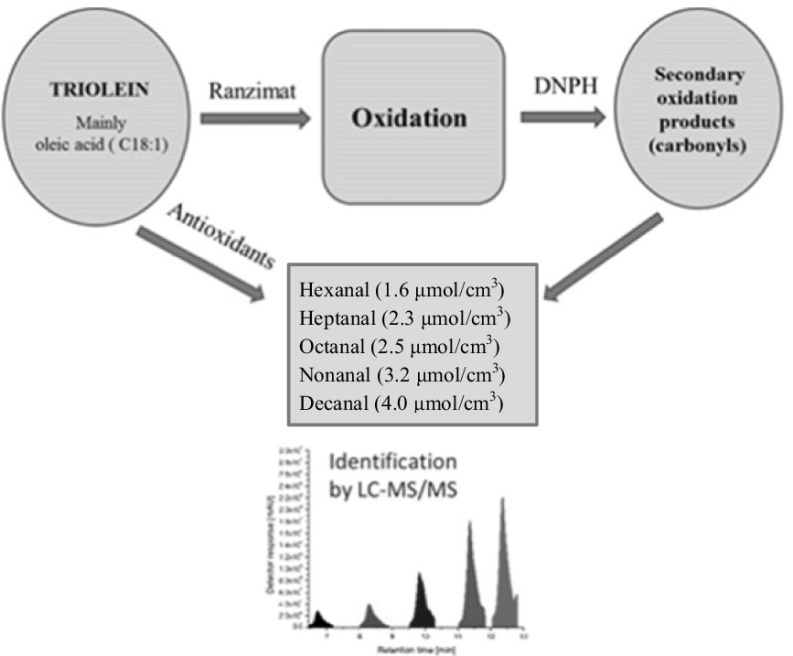

## Introduction

Prolonged exposure to oxidized lipids can be detrimental to human health. It has been reported that lipid hydroperoxides from processed dietary oils can enhance the growth of hepatocarcinoma cells [1]. Another issue was published by Böhm and co-workers [2] who found that oxidized fat might result in liver inflammation which was shown in experimental rats. In the latter publication, no relation to a specific substance or group of substances was established. Therefore, a quantitative characterization of oxidized oils is a prerequisite to start further mechanistic investigations on the toxic principles of oxidized oils.

In earlier manuscripts the formation of different peroxides (hydroperoxides, epidioxides) and epoxides was described in detail [3, 4]. A second group of reactive products from oil oxidation are the carbonyls which are formed during further reactions of the primary peroxides.

The formation of carbonyls during oil oxidation was investigated earlier in rapeseed oil [5] when it was shown that as a result of the degradation of the intermediate hydroperoxides a great variety of aldehydes and ketones are formed. It is well known that medium and short chain aldehydes which are formed from higher unsaturated fatty acids are the key aroma-active components which are responsible for a rancid aroma. These are C_7_–C_11_ monounsaturated aldehydes, or C_6_–C_9_ dienals, or C_5_ branched aldehydes or some C_8_ ketones which are important contributors to the oil aroma having negative attributes (rancid, winey-vinegary, fusty, muddy sediment, musty) [6].

 These carbonyls and other compounds such as aldehydes, terminal alkenes, carboxylic acids, and aliphatics are formed by a β-cleavage of lipid alkoxyl radicals [7]. The fragmentation reactions of allylic hydroperoxides by the Hock rearrangement create two different carbonyls from the same lipid peroxide, i.e. aldehydes or ketones [8]. Mechanistically, this is achieved by the insertion of one oxygen atom into the lipid carbon backbone and delocalization of a positive charge on a carbon or oxygen atom.

The main aim of this work was to establish an analytical method for a quantitative determination of the carbonyls in the lipid (oxidized oil) phase for determination of the alimentary exposure. For the easier identification and assignment of the reaction products, a defined matrix (triolein) which did not contain any interfering compounds was used. It was decided to use the derivatization with 2,4-dinitrophenylhydrazine (DNPH)—a commonly known derivatization reagent for carbonyls—that would give the possibility to identify and quantify single components as well as the total carbonyl content. These derivatives are stable and can be analysed by HPLC with ESI-MS in the negative mode or by its absorption at 400 nm [9–11].

With this background, we have started to characterize the formation of carbonyls during oxidation of triolein in the oil phase and not the volatile phase in which these compounds are mostly related to sensory sensation. On basis of reversed phase chromatography with UV and MS detection, the non-evaporating carbonyl compounds formed during oxidation of triolein were identified and quantified. In this case, triolein was used as model oil. The results of these experiments will be a basis for future work on food oil oxidation. In addition, the interaction of antioxidants (β-carotene, α-tocopherol) with the carbonyl formation was analysed.

## Results and discussion

### Secondary oxidation products analysis

**Fig. 1:**
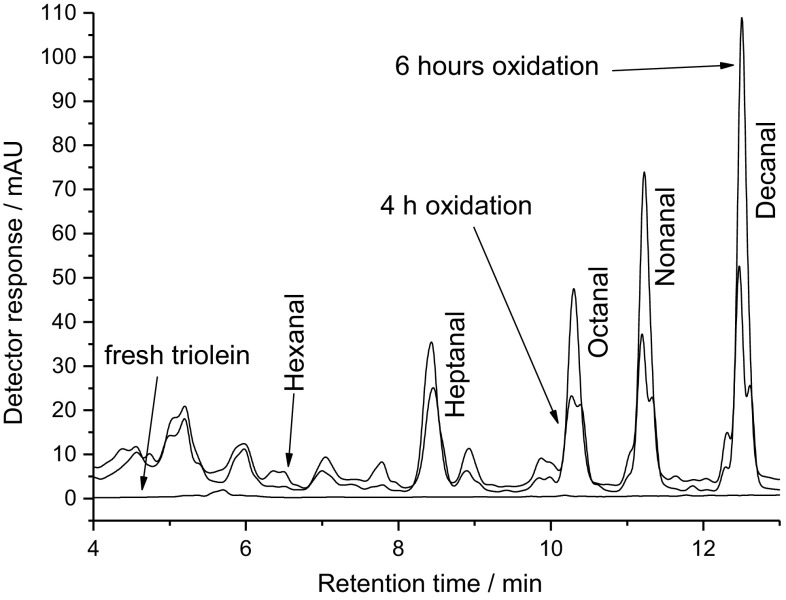
Chromatogram of DNPH derivatives of fresh triolein and after 4 h as well as 6 h of oxidation in the Rancimat

The derivatization of carbonyls is commonly used in chromatographic analysis. The hydrazones formed are stable enough for MS experiments. In some cases the carbonyls from fatty acid oxidation are too volatile to be analysed by LC/MS. Therefore, the DNPH adds molecular weight to increase sensitivity and selectivity [12]. Negative ionization using APCI was the most sensitive MS method which could be used. The hydrazones formed were analysed using visible light absorption (400 nm) or by MS. The advantage of detection at 400 nm is that practically all peaks that appeared in the chromatogram were DNPH derivatives. In the control chromatograms only the baseline could be observed at 400 nm (Fig. 1). The semiquantitative evaluation of the carbonyls was based on a calibration using hexanal with the peak area relating to the molar concentration of hexanal. The linear calibration of hexanal was done in the range of 8–1000 µg/cm^3^ (0.08–10 mM). The formation of carbonyls could be explained by a Hock cleavage which was reviewed by [7]. This might explain the formation of, e.g. decanal and 2-undecenal which are produced by homolytic cleavages on either sides of the 8-hydroperoxide and nonanal from either the 9- or 10-hydroperoxide. The linearity of the hexanal analysis was tested in the range of 5–1300 µg/cm^3^ according to Mandel [13] with a limit of detection of 4.1 µg/cm^3^ and a limit of quantification of 13 µg/cm^3^.

The formation of carbonyls began after 2 h of oxidation at 120 °C while bubbling air through the oil. A strong increase of carbonyl formation was observed between 2 and 4 h with a maximum after 6 h and a slight decrease or constant concentrations afterwards. A series of linear aldehydes (from 6 carbons to 10 carbons) could be identified positively by LC–MS. Based on the calibration with hexanal all other dominant substances were in the similar concentration range with maximum concentrations of 1.6 µmol/cm^3^ of hexanal, 2.3 µmol/cm^3^ of heptanal, 2.5 µmol/cm^3^ of octanal, 3.2 µmol/cm^3^ of nonanal, 4.0 µmol/cm^3^ of decanal after 6 h (Fig. 4). These identified aldehydes were volatile and undergo further degradation reactions which may explain the reduced concentration.

The double peaks which could be seen after 4 h of oxidation were attributed to *Z*/*E* isomer formation since no difference in the mass spectrum and UV absorption could be observed. After 6 h of oxidation the double peaks disappear resulting in a shift in the relative concentrations (Fig. 1).

**Fig. 2:**
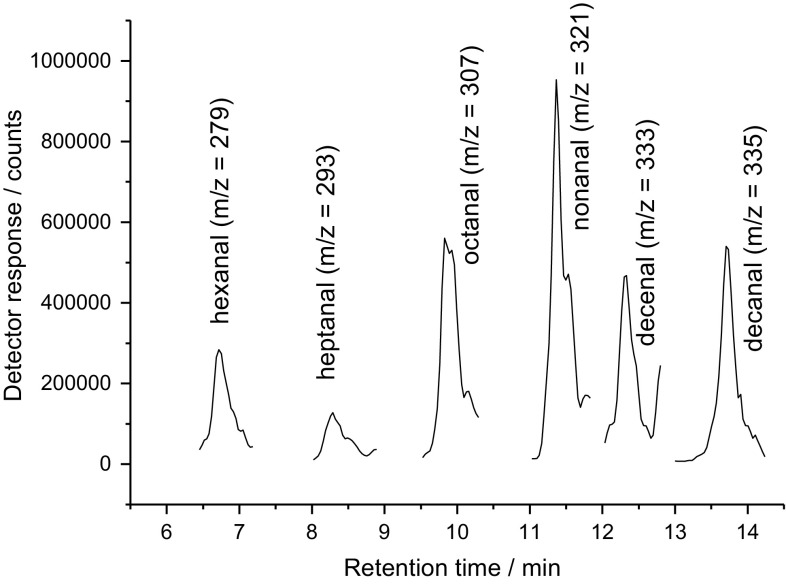
Selected ion traces of the DNPH conjugates of the saturated and unsaturated aldehydes found in oxidized triolein

The identification of the peaks was done by LC–MS/MS as is shown in Fig. 2. The earlier mentioned saturated aldehydes (hexanal–decanal) including decenal could be identified. The fragmentation of all of these compounds was similar with a specific fragment of *m*/*z* = 182 originating from the DNPH moiety.

In this experiment, nonanal and octanal were the main products. 2-Nonenal and 2,4-decadienal which are the known degradation products of linoleic acid were not detected. Additional compounds (heptane, heptanol, octane, 1-undecene, and 2-undecenal) that were described earlier are produced by a homolytic fission of the intermediary formed R–O bond [14–19]. However, non-carbonyls could not be detected with the method described here.

**Fig. 3:**
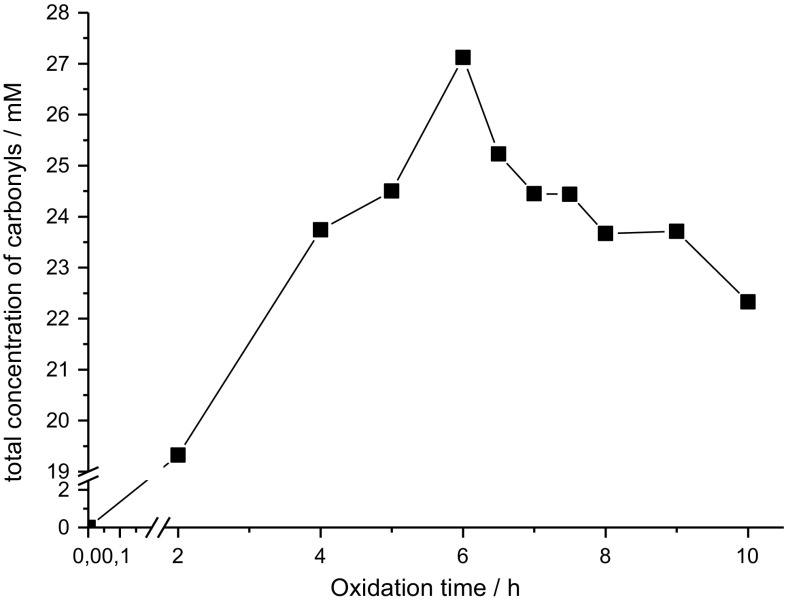
Formation of the total amount of carbonyls during oxidation of triolein at 120 °C with constant air sparging

The total amount of carbonyls formed is shown in Fig. 3. Similar to the single aldehydes, a maximum concentration is reached after 6 h with a decrease afterwards. The maximum concentration is 27 mM which is based on a calibration with hexanal.

### Influence of lipid-soluble antioxidants on carbonyl formation

In a second series of experiments, two lipid-soluble antioxidants were added, namely α-tocopherol and β-carotene. The earlier experiments showed that the best stabilization was obtained by addition of 300 ppm of ascorbyl palmitate to soybean oil (SO). In addition, a combination of ascorbyl palmitate (300 ppm) and α-tocopherol (1000 ppm) was able to limit hydroperoxide and hexanal formation in SO at 35 °C for 12 weeks [20].

**Fig. 4:**
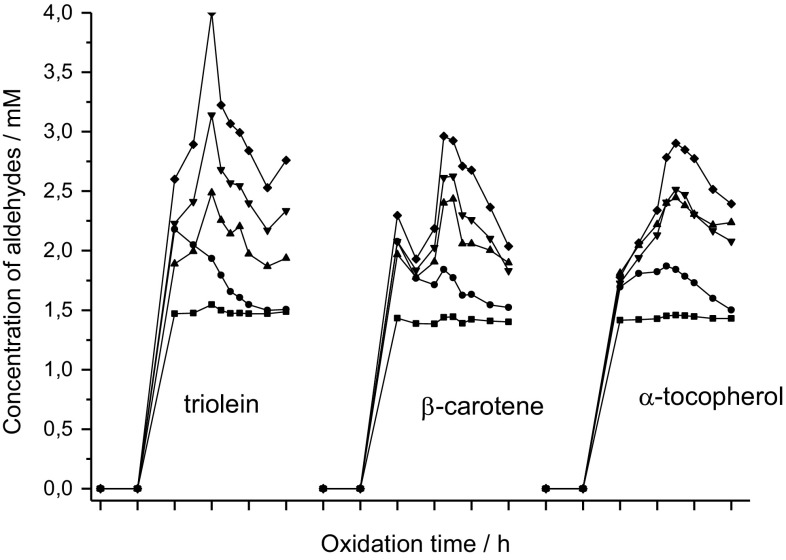
Formation kinetics of single compounds during oxidation of triolein in presence of lipid-soluble antioxidants at 120 °C with constant air sparging for 10 h (*filled circle* hexanal; *filled square* heptanal; *filled upward triangle* octanal; *filled downward triangle* nonanal; *filled diamond* decanal)

**Fig. 5:**
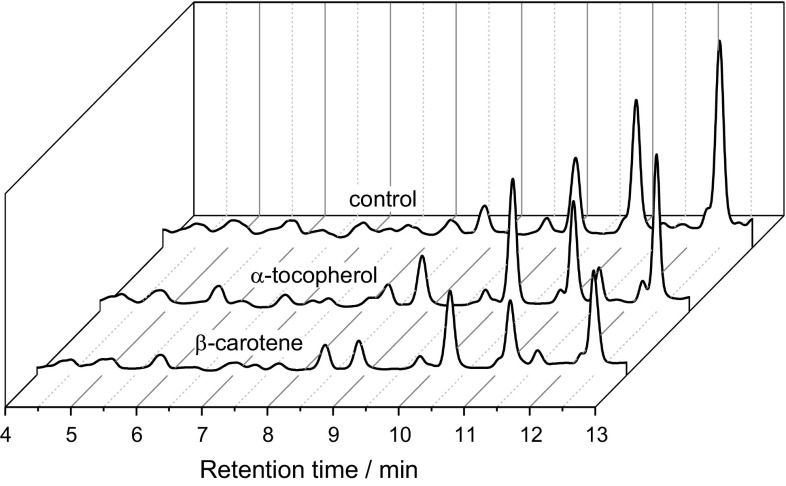
Comparison of the carbonyl formation in presence of lipid-soluble antioxidants (α-tocopherol, β-carotene) after 10 h of oxidation at 120 °C. The carbonyls are measured as DNPH derivatives detected at 400 nm

Antioxidants inhibit lipid oxidation by quenching free radicals and physically stabilizing the micelles at reaction sites [21]. As described in [22] the oxidation starts after 5 h in both, the refined and fortified oils (containing β-carotene), with the formation of hydroperoxides. In this case, the addition of β-carotene resulted in a pro-oxidant effect. β-Carotene was still measurable after 10 h [22]. During the course of the oxidation in the experiments described here, the levels of the formed carbonyls were lower in the samples with added antioxidants (Fig. 4). In addition, the pattern of the chromatograms did not change significantly indicating the same carbonyls are formed but at lower amounts. The sample containing α-tocopherol showed a higher formation of octanal after 10 h (Fig. 5).

For this, β-carotene and α-tocopherol were dissolved in acetone and added to the triolein. The concentrations of the antioxidants were 300 µg/g ± 0.5 µg. The fortified triolein was stirred for 15 min and then flushed with nitrogen for 30 min to remove the acetone before sealing airtight in glass bottles. For the oxidation in the Rancimat 20 g of triolein was used. The concentrations which were applied were in the same range as it can be expected in natural oils. The α-tocopherol concentration was 300 µg/g and the level of β-carotene was 500 µg/g. The concentrations were selected according to the concentrations normally occurring in red palm oil which are in the range of 600–1000 µg/g for vitamin E and 400–3500 µg/g for α- and β-carotene [23].

## Conclusion

The formation of carbonyls was quantified during oxidation of triolein by derivatization of the formed products with DNPH which is a selective reagent for aldehydes and ketones. Some of the oxidized fragments of triolein were identified by LC–MS/MS—using APCI in negative mode—being hexanal (*m*/*z* = 279), heptanal *(m/z* = 293), octanal (*m/z* = 307), nonanal (*m*/*z* = 321), decenal (*m*/*z* = 333), decanal (*m*/*z* = 335), and undecenal (*m*/*z* = 347). Using a Rancimat for reproducible oxidation experiments with a constant air flow at defined temperatures (120 °C) the production of carbonyls showed a good repeatability. The formation of carbonyls from triolein showed a maximum after 6 h with a slight or no decrease during prolonged oxidation.

## Experimental

2,4-Dinitrophenylhydrazine (2,4-DNPH) was purchased from Sigma-Aldrich (St. Louis, USA), hydrochloric acid (HCl, 37%) was purchased from Merck (Darmstadt, Germany), all solvents (e.g. methanol, ACN, acetone) used were of HPLC grade and were purchased from Merck (Darmstadt, Germany), acetic acid was purchased from Roth (Karlsruhe, Germany), triolein was purchased from FLUKA (Buchs, Switzerland), β-carotene and α-tocopherol were from Sigma-Aldrich (St. Louis, USA).

### Analysis of secondary oxidation products in oil

#### Oxidation of triolein with Rancimat

The triolein oil samples were subjected to oxidation in a Rancimat (679, Metrohm, Herisau, Switzerland). Eleven grammes of sample was used for the oxidation experiments. The temperature was set to 120 °C and the air flow to 20 dm^3^/h. Triolein was treated for up to 10 h. The oxidized samples were cooled immediately after the oxidation and stored under nitrogen below −18 °C.

#### Derivatization with 2,4-dinitrophenylhydrazine (DNPH)

To 1 cm^3^ of the oxidized oil samples 4 cm^3^ of acetonitrile was added and mixed with 3 cm^3^ of the reagent 2,4-DNPH (3.48 mg/cm^3^). The reaction mixture was kept in the dark for 1 h. After completion of the reaction, 2 cm^3^ ethyl acetate was added for extraction and 1 g KCl for better phase separation. This mixture was thoroughly shaken for 30 s and centrifuged for phase separation. The organic layer was analysed without further treatment by HPLC.

#### Liquid chromatography–mass spectrometry condition for aldehydes identification

The analyses of the DNPH derivatives of the carbonyls formed during oxidation were done by HPLC (Agilent 1100, Waldbronn, Germany) using a reversed phase column (Kinetex, EVO C18, 150 × 3 mm, 5 µm, Phenomenex, Aschaffenburg, Switzerland). For elution a gradient was used starting with methanol (45%), water (30%), and acetonitrile (25%) changing to methanol (6%), water (4%), and acetonitrile (90%) linearly within 15 min. The absorption of the eluent was measured at 400 nm for the presence of the DNPH derivatives.

For mass selective detection, a QTRAP 2000 (AB Sciex, Framingham, MA, USA) was used. Ionization was done using the APCI mode with a gas drying temperature of 250 °C, capillary voltage of 4000 V, and a fragmentor potential of 150 V.

### Analyses of α-tocopherol and ***β***-carotene

For the analyses of α-tocopherol and β-carotene 25 mg of the oil (triolein, triolein with β-carotene and triolein with α-tocopherol) was extracted with 1 cm^3^ of methanol in 2-cm^3^ reactions vials (Eppendorf, Wien, Austria). The samples were shaken for 2 min vigorously and centrifuged. Under these conditions, both α-tocopherol and β-carotene were extracted quantitatively. The methanolic extract was used directly for HPLC analysis on a reversed phase column (Kinetex, EVO C18, 150 × 3 mm, 5 µm, Phenomenex, Aschaffenburg, Switzerland) using a flow of 0.6 cm^3^/min. α-Tocopherol was separated isocratically using 5% water in methanol detecting it at 292 nm. β-Carotene was chromatographed with 100% acetonitrile with detection at 450 nm.

## References

[1] Rohr-Udilova NV, Stolve K, Sagmeister S, Nohl K, Schulte-Hermann R, Grasl-Kraupp B (2008). Mol Nutr Food Res.

[2] Böhm T, Berger H, Nejabat M, Riegler T, Kellner F, Kuttke M, Sagmeister S, Bazanella M, Stolze K, Daryabeige A, Binther N, Murkovic M, Wagner KH, Schulte Hermann R, Rohr-Udilova N, Huber W, Grasl-Kraupp B (2013). J Hepatol.

[3] Zeb A, Murkovic M (2013). Food Res Int.

[4] Zeb A, Murkovic M (2011). Food Chem.

[5] Farhoosh R, Pazhouhanmehr S (2009). Food Chem.

[6] Angerosa F (2002). Eur J Lipid Sci Technol.

[7] Frankel EN (2005). Lipid oxidation.

[8] Schneider C, Porter NA, Brash AR (2008). J Biol Chem.

[9] Schulte E (2002). Anal Bioanal Chem.

[10] Mottram HR, Woodbury SE, Evershed RP (1997). Rapid Comm Mass Spectrom.

[11] Ochs S de M, Fasciotti M, Netto ADP (2015). J Spectrosc.

[12] Potter W, Karst U (1996). Anal Chem.

[13] Mandel J (1984). The statistical analysis of experimental data.

[14] Feron VJ, Til HP, de Vrijer F, Woutersen RA, Cassee FR, van Bladeren PJ (1991). Mutat Res.

[15] Qu YH, Xu GX, Zhou JZ, Chen TD, Zhu LF, Shields PG, Wang HW, Gao YT (1992). Mutat Res.

[16] Ruiz-Méndez MV, Aparicio R, Harwood J (2003). Refining of olive and pomace oils. Olive oil manual.

[17] Subramanian R, Nakajima M (1997). J Am Oil Chem Soc.

[18] Wan PJ, Pakarinen DR, Miscella RJ (1996). J Am Oil Chem Soc.

[19] Katragadda HR, Fullana A, Sidhu S, Carbonell-Barrachina AA (2010). Food Chem.

[20] Sarkar A, Golay PA, Acquistapace S, Craft BD (2015). Int Food Sci Technol.

[21] Budilarto ES, Eldin AK (2015). Eur J Lipid Sci Technol.

[22] Murkovic M, Wiltschko D, Pfannhauser W (1997). Fett/Lipid.

[23] Ooi CK (1999). PORIM Bull.

